# Experimental verification and PK/PD modeling of selective drug absorption via acupoint administration in rabbit model of rheumatoid arthritis

**DOI:** 10.3389/fneur.2026.1754856

**Published:** 2026-06-19

**Authors:** Zhoubo Yu, Jing Yang, Junhao Zhang, Yuxin He, Ming Liu, Pengfei Ren, Jiannan Meng, Rui Wang

**Affiliations:** 1 College of Pharmacy, Heilongjiang University of Chinese Medicine, Harbin, Heilongjiang, China; 2 Basic Medical College, Heilongjiang University of Chinese Medicine, Harbin, Heilongjiang, China; 3 Key Laboratory of Basic and Application Research of Beiyao, Heilongjiang University of Chinese Medicine, Ministry of Education, Harbin, Heilongjiang, China

**Keywords:** acupoint, cationic liposome, microneedle, miRNA55, PK/PD modeling, rheumatoid arthritis, transcutaneous

## Abstract

**Introduction:**

The administration of drugs at specific acupoints is a traditional practice in Chinese medicine, yet scientific evidence supporting its pharmacokinetic and pharmacodynamic (PK/PD) advantages remains limited. This study aimed to evaluate the transdermal absorption and therapeutic effects of miRNA55 when delivered via microneedles at different acupoints in a rabbit model of rheumatoid arthritis (RA).

**Methods:**

An RA model was established in New Zealand rabbits using ovalbumin-Freund’s complete adjuvant emulsion. miRNA55 was encapsulated in cationic liposomes and combined with hyaluronic acid for microneedle preparation. Microneedles were applied at classical acupoint (ST36), atypical acupoints (GB34), and non-acupoint areas above the knee joint of the rabbits (avoiding ST35). Drug concentrations in the joint cavity fluid were assessed by microdialysis and enzyme-linked immunosorbent assay (ELISA). The encapsulation rate and *in vitro* release of miRNA55 were analyzed by UV spectrophotometry. PK/PD modeling was conducted using WinNonlin 8.1, with IL-1β, TNF-*α*, and IL-6 as pharmacodynamic markers. The protein expression of TNF-α, MMP-1, and MMP-3 was assessed using western blotting (WB), and histopathological changes in the synovial tissue were observed by hematoxylin–eosin (H&E) staining.

**Results:**

miRNA55 concentrations in the joint cavity were the highest in the ST36 group, followed by the GB34 and non-acupoint groups. This observation indicated that the degree of drug absorption. PK/PD modeling showed higher efficacy value for ST36 across all three cytokines. For IL-1β, Emax model for ST36 was $\;E=\frac{81.09C_{e}}{169.56+C_{e}}$ ; for TNF-*α*, $E=\frac{70.35C_{e}}{136.15+C_{e}}$ ; and for IL-6, $E=\frac{69.93C_{e}}{167.88+C_{e}}$ , all higher than those observed in the GB34 and non-acupoint groups. WB analysis confirmed lower expression level of inflammatory proteins in the ST36 group. H&E staining showed reduced synovial erosion and lymphokine infiltration in the ST36 group compared to those in the other two groups.

**Discussion:**

This study demonstrates that transdermal administration of miRNA55 via microneedle at specific acupoints enhances drug absorption and improves therapeutic outcomes in a rabbit model of RA. These findings support the traditional concept of acupoint specificity and offer a scientific basis for integrating acupoint-based drug delivery in modern RA treatment strategies.

## 1 Introduction

Rheumatoid arthritis (RA) is a chronic autoimmune disease that manifests as inflammation of the synovial joints. RA involves a systemic inflammatory response, affecting all organs of the human body and resulting in bone deformities and death (1). Globally, RA affects 0.5–1% of the population, with significantly higher rates in Western countries and a three times greater occurrence in women than in men. Although the pathogenesis of RA is not yet clear, genetic, dietary, and environmental factors are known to contribute to its development (2). In traditional Chinese medicine (TCM), RA is categorized under the syndrome of “Bi Zheng” (3).

Current first-line clinical treatments for RA include oral non-steroidal anti-inflammatory drugs (such as aspirin and ibuprofen), antirheumatic drugs (methotrexate), glucocorticoids, dexamethasone, etc. (4). While these treatments offer symptom relief and slow disease progression, they are associated with significant side effects, such as gastrointestinal complications, cardiovascular and hepatopulmonary toxicity, and ulcers. Glucocorticoids can also cause osteonecrosis and atherosclerosis (5). Therefore, researchers are increasingly interested in developing safer and more targeted therapeutic approaches, including those derived from TCM.

MicroRNAs (miRNAs) are small, non-coding RNA molecules that regulate gene expression and cellular function. miRNAs have also been implicated in autoimmune diseases, including RA. Increased levels of miR-155 and miR-146a, along with decreased expression of miR-142-3p, have been observed in patients with RA, highlighting their potential as biomarkers and therapeutic targets. In addition, miRNA-143 and miRNA-145 have been shown to enhance miRNA-targeted therapy in RA-fibroblast-like synoviocytes (RA FLSs) and are increasingly used to alleviate RA (6). miRNA55, extracted from a TCM prescription for removing stasis under the diaphragm, has shown promise in modulating inflammatory responses in preclinical studies (7).

Microneedles (MNs) are drug delivery systems consisting of a number of thin (25–1,000 μm) needle tips that create microchannels on the skin surface to improve drug absorption and minimize systemic side effects. While traditional microneedles are at risk of sharp waste and tip breakage, soluble MNs are made of biodegradable materials, such as hyaluronic acid (HA) and dissolves when inserted into the skin, eliminating the risk of sharp waste and providing contact with the skin interstitial fluid, eliminating the possibility of secondary infections caused by the repeated use of MNs (8). HA has the characteristics of non-toxicity, non-immunogenicity, biocompatibility, and high-water affinity and can interact with the cuticle barrier and promote drug penetration (9). Additionally, liposomes, used as non-viral vectors, do not cause infection, have no carrier capacity constraint, possess controllable chemical structure, have no tumorigenic risk or antigenicity, and can be loaded with both water-soluble and fat-soluble substances (10).

In TCM theory, the internal conditions and disorders of organs are reflected at specific points located on the surface of (or under) the skin, and these points are often called acupoints. Drug administration via acupoints is believed to enhance therapeutic effects by regulating organ function, maintaining homeostasis, and treating diseases (11). As drug accumulates at the administration site, it stimulates the acupuncture points and increases the skin temperature, which in turn increases transdermal absorption of the drug. Elevated skin temperature also dilates the capillaries and promotes the drug entry into the meridians and systemic distribution (12). This method allows direct delivery to the disease site, bypasses the liver’s first-pass effect of drugs, maintains stable drug concentration in blood, and may improve patient compliance (13).

The PK-PD model uses mathematics to describe biological and pharmaceutical processes as much as possible, providing unique advantages for the study of drug mechanisms and actions (14). Moreover, it integrates drug concentration with its pharmacological effect, providing a comprehensive understanding of drug action.

This study aimed to bridge traditional acupoint-based TCM therapy with modern drug delivery technology and provide new insights into RA treatment strategies. Toward this, miRNA55 was loaded into cationic liposomes combined with HA to prepare soluble MNs. The formulation was administered transdermally at different acupoints in a rabbit model of RA. PK/PD analysis, histopathological evaluation, and inflammatory protein expression were performed to assess the therapeutic effects and explore the influence of acupoint selection on drug absorption.

## 2 Materials and methods

### 2.1 Experimental drug

FAM-miRNA55 was encapsulated in cationic liposomes composed of DOTAP and cholesterol (CHOL), which were loaded in HA-MNs. Each microneedle patch, fabricated in-house, contained 33.3 ± 2.98 μg of miRNA55. The MNs had a tip length of 500 μm and a patch size of 1.5 cm × 1.5 cm.

### 2.2 Animals

Male New Zealand rabbits (2.5–3.0 Kg) were provided by the Experimental Animal Center of Heilongjiang University of Chinese Medicine (experimental animal qualification certificate number: SYXK (Black) 2022-001). Animals were housed under controlled conditions (24.0 ± 2.0 °C, 50% relative humidity). The research equipment was disinfected regularly; the light and dark cycle was maintained for 12 h. Animals were provided standard feed and tap water. After a 7-day acclimation period, animals were used for the study. The study strictly followed the ethical code of Heilongjiang University of Chinese Medicine for the management and use of laboratory animals. All animal experiments were conducted in accordance with the regulations of the Animal Ethics Committee of the School of Chinese Medicine, Heilongjiang University (No. 2020101601).

### 2.3 Reagents

Ovalbumin (relative molecular mass 45,000) (Shanghai Boao Biotechnology Co., LTD., lot No. 020902); Freund’s complete adjuvant (SIGMA Corporation, lot No. 210112); HA (Shandong Keyuan Biochemical Co., LTD., 400000Dka, lot No: C16991179); pentobarbital sodium (Shanghai Xingzhi Chemical Plant, lot No. 921019); heparin sodium (Jiangsu Wanbang Pharmaceutical Co., LTD., lot No. 211022B); rabbit TNF-*α*; interleukin (IL)-1β, IL-6, and enzyme-linked immunosorbent assay (ELISA) kits (Nanjing Institute of Biological Engineering, Batch No. 20211220).

### 2.4 Instruments and equipment

CNC ultrasonic cleaner (KQ5200DE, Kun Shan Ultrasonic Instruments Co., Ltd.); magnetic stirrer with constant temperature heating (Zhengzhou Greatwall Scientific Industrial and Trad); electronic analytical balance (AB265-S, Shanghai liangping Instrument Co., Ltd.). Eddy oscillator (MS3 Basic; IKA, Germany), CMA20EliteMicrodialysisProbe4mm (CMA Corporation of Sweden); TubingAdapter (CMA Corporation of Sweden); CMA402 dual channel automatic collector (CMA Corporation of Sweden); Joint microdialysis probe CMA/20 (CMA Corporation of Sweden); Perfusion device CMA1.0 mL (CMA Corporation of Sweden); Microdialysis pipeline FEPTubing1M (CMA Corporation of Sweden); Microplate Reader (Synergy H1; Botten Instruments Co., Ltd.); and high-speed refrigerated centrifuge (Tg-16 m; Shanghai Luxiang Yi Centrifuge Instrument Co., Ltd.).

### 2.5 Animal model of RA

The RA model was established as previously described (15). Briefly, the villi on the shoulder blades and knee joints of the lower limbs were removed and cleaned with a hair-removal agent, and the hair-removal area was cleaned with a 75% ethanol solution. After the rabbits were anesthetized with pentobarbital sodium, RA was induced in rabbits by subcutaneous injection of 1 mL of an emulsion containing 20 mg/mL ovalbumin and 10 mg/mL Freund’s Complete adjuvant (15) at five points on the back. This sensitization was repeated weekly (every 7 days) for 3 weeks. In the fourth week, both sides of the suspensor ligament in the middle of the line between the highest point of the tibial tubercle at the knee joint and the lower margin of the patella were selected as injection points. The needle tube was placed perpendicular to the skin, and 1 mL of Ovalbumin liquid solution was injected subcutaneously to strengthen and induce RA.

### 2.6 Preparation of miRNA55-loaded cation liposomes (CLs)-HA microneedles

miRNA55 was dissolved in 0.1% DEPC-treated water and mixed with an appropriate amount of the cationic lipid DOTAP and the co-lipid CHOL. The mixture was incubated at room temperature for 30 min; miRNA55 was promoted into liposomes through spontaneous electrostatic interactions. Simultaneously, HA was dissolved in 0.1% DEPC-treated water, and air bubbles were removed under vacuum. The miRNA55-CLs were then mixed with HA solution and cast into molds. After drying, the miRNA55-CLs-MNs were obtained.

### 2.7 Transdermal drug release of miRNA55 HA-MNs *in vitro*

*In vitro* transdermal drug release experiments were conducted using Franz diffusion cells (16). HA-MNs loaded with miRNA55 were applied to hairless rat abdominal skin, which was mounted in the donor compartment of the diffusion cell with the cuticle layer facing up. The Franz diffusion cell was placed in a 0.1% DEPC constant temperature water bath at 37.5 ± 0.5 °C, and the stirring speed was 100 rpm/min. Samples were collected at predetermined intervals from the recipient chamber, and a fresh buffer solution was used as a supplement.

### 2.8 Determination of microneedle encapsulation rate

The miRNA55-CLs-HA-MNs were dissolved in 0.1% DEPC-treated water in an ice bath and then subjected to ultrasonic disruption. The optical density (OD) of the resulting solution was measured at 492 nm using an enzyme-labeled microplate reader. The encapsulation rate of miRNA55 was determined based on the absorbance values (17).

### 2.9 Experimental groups and administration

Kuo et al. reported that ST36 was used to treat knee RA by 101 and GB34 by 84 studies (18). Therefore, ST36 and GB34 were selected as the acupoints for percutaneous administration in the current study. Non-acupoint administration above the knee joint (avoiding ST35) was selected. Liyan et al. have previously determined the acupuncture points in rabbits and administered drugs (19), and we used the same approach. Eighteen rabbits with successfully induced RA model were randomly divided into blank, ST36 percutaneous, GB34 percutaneous, and non-acupoint percutaneous groups, with six rabbits in each group. The drug administration group was performed according to animal body mass (miRNA55 content per microneedle was 33.3 ± 2.98 μg). The blank group was administered blank MNs. Microneedle patches (1.5 cm × 1.5 cm) were affixed to designated acupoint or non-acupoint sites and secured with non-fat gauze and tape for 14 h.

### 2.10 Group treatment and determination of miRNA55 in the joint cavity

Rabbits with successfully established RA were fasted for 24 h before the experiment while maintaining free access to drinking water. After anesthetization using pentobarbital sodium, rabbits were fixed on the operating table with their abdomens facing up. The blank model group received no drug treatment. The acupoint administration group was administered the drug according to the established administration route, and the nonacupoint administration group received miRNA55 MNs applied to specific acupoints (ST36 or GB34), while the non-acupoint group received MNs at a site proximal to the knee joint, avoiding ST35. Microdialysate samples from the joint cavity were collected every hour for a total of 14 h. The samples were analyzed using enzyme markers, and the data were recorded. The results are summarized in Table 1. No detectable drug was observed in samples from the blank model group. The recovery rate of the probe was measured using the zero-net flux method.

**Table 1:** Changes in miRNA55 content in the articular cavity dialysate with different routes of administration (*n* = 6).

Time (h)	ST36	miRNA55 (ng/mL)	Non-point
		GB34	
0.	0	0	0
1.	66.94 ± 4.32	58.59 ± 6.26	63.49 ± 5.33
2.	129.68 ± 5.89	91.41 ± 7.53	125.24 ± 6.36
3.	152.25 ± 4.34	119.66 ± 8.26	149.38 ± 8.56
4.	168.93 ± 5.70	145.57 ± 5.66	159.63 ± 7.59
5.	188.75 ± 4.95	160.09 ± 6.24	152.21 ± 6.23
6.	192.64 ± 7.57	169.85 ± 6.26	106.95 ± 7.26
7.	183.78 ± 8.56	159.19 ± 7.27	96.51 ± 5.21
8.	179.14 ± 6.53	153.28 ± 8.28	113.21 ± 7.99
9.	175.33 ± 5.03	133.65 ± 7.27	101.08 ± 5.03
10.	169.39 ± 4.37	135.25 ± 6.56	103.20 ± 6.31
11.	154.77 ± 4.33	119.06 ± 6.86	100.11 ± 6.89
12.	148.20 ± 5.94	106.55 ± 7.53	102.79 ± 5.21
13.	132.71 ± 4.88	101.87 ± 5.38	89.32 ± 5.00
14.	127.36 ± 5.09	92.46 ± 5.90	82.90 ± 4.53

### 2.11 Pharmacokinetics of miRNA55 MNs

PK analysis of miRNA55 following administration via different routes (ST36, GB34, and non-acupoint) was conducted using WinNonlin Examples Guide software (version 8.1). The minimum information criteria (AIC) and Bayesian information criteria (SBC) were calculated to infer the attribution of the atrioventricular model and to calculate and analyze the pharmacokinetic data fitting.

### 2.12 Pharmacodynamics of miRNA55 MNs

To evaluate PD effects, IL-1β, TNF-*α*, and IL-6 were selected as immunohistochemical indexes for pharmacodynamic study, and miRNA55 micro-targeted immune indexes were investigated using ELISA. The effect (E) of IL-1β, TNF-α, and IL-6 is the inhibition rate (IR) of miRNA55 against IL-1β, TNF-α, and IL-6, where $\;\mathrm{IR}=\frac{\left(C_{0}-C_{T}\right)}{C_{0}}\times 100\%$ . Where C0 is the initial mass concentration, and Ct is the post-treatment concentration.

### 2.13 Selection of PK/PD model

### 2.14 Study on local PK/PD model of joint cavity

The mass concentration-time of miRNA55 in the joint cavity was imported into WinNonlin software (version 8.1) to establish a PK/PD model. Using IL-1β, TNF-α, and IL-6 as pharmacodynamic indexes, the mass concentration-effect curve was generated for each active component in the effect chamber. After fixing the PK parameters, the time-effect relationship was fitted by software to obtain PD parameters. The PK/PD binding model parameters of miRNA55 were obtained, and a PK/PD model was established based on these parameters. Finally, all the parameters were substituted into the corresponding pharmacodynamic model equation to obtain the pharmacodynamic equation.

### 2.15 Hematoxylin–eosin pathological staining

The synovial tissue of the ankle in each group was fixed in 4% paraformaldehyde for 48 h. Tissues were dehydrated in graded ethanol (80, 90, 95, and 100%) for 2 h each, followed by xylene clearing and paraffin embedding. Sections were sliced and stained using the H&E method. Histopathological changes were observed under a light microscope (DM2500, Leica, Germany), and images were collected. Histopathological changes in rabbit ankle joints were evaluated in a blinded manner. A semi-quantitative scoring system was used to assess four key parameters: synovial hyperplasia, inflammatory cell infiltration, pannus formation, and cartilage and bone destruction. Each parameter was scored from 0 to 4, yielding a total histopathological score ranging from 0 to 16. For each section, five randomly selected high-power fields (×100 magnification) were examined, and the mean score was used for statistical analysis.

### 2.16 Statistical analysis

The data obtained were analyzed and processed using GraphPad 8.0.2 statistical software (GraphPad Software, San Diego, California), and the measurement data are expressed as means $\bar{x}$ ±s standard deviations. One-way analysis of variance was used for comparison between groups. *p* < 0.05 indicated that the difference was statistically significant.

## 3 Results

### 3.1 Stimulate the temperature change of specific parts

### 3.2 Successful induction of RA model

Following model establishment, rabbits in the model group exhibited significantly reduced activity, decreased food intake, and elevated skin surface temperature compared to those from the blank group. Over time, the body temperature gradually returned to the normal but progressive signs of joint inflammation were observed. These symptoms included joint redness, fever, joint swelling. Severe joint swelling led to altered gait pattern and reduced range of motion. Joint manipulations tended to lead to resistance in some animals and was accompanied by stiffness. Quantitative measurements confirmed joint swelling. For example, the joint diameter in the model group increased to approximately three times that of the blank group (The total volume of the lower limb knee joint). The swelling later stabilized to approximately twice the diameter of the blank group. These findings indicate successful induction of an inflammatory arthritis model.

### 3.3 Microneedle appearance and performance

**Figure 1:**
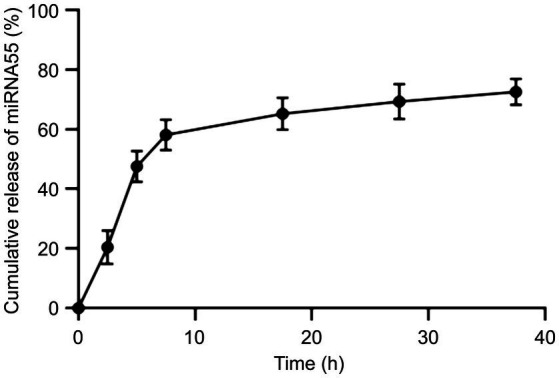
*In vitro* release profile of miRNA55 from hyaluronic acid microneedles.

**Figure 2:**
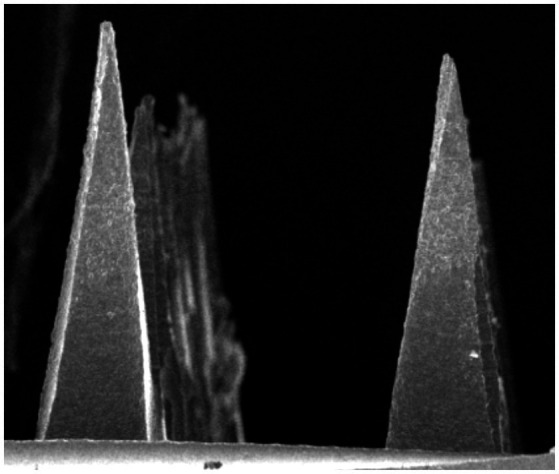
Scanning electron microscopy images of the microneedle patch (scale bar = 500 μm).

*In vitro* drug release from the miRNA55-loaded HA-MNs (miRNA55-HA-MN) followed a biphasic profile (Figure 1). An initial exponential release phase occurred within the first 8 hours, during which approximately 60% of miRNA55 was released, followed by a slower sustained release. These results indicated that the HA matrix facilitated the efficient transdermal delivery and prolonged drug retention in the skin. The encapsulation efficiency of miRNA55 within the HA MNs was determined to be 91.4 ± 2.03%, indicating successful loading of the therapeutic agent into the microneedle system. SEM imaging revealed that MNs were uniformly shaped with a conical structure and tip of approximately 500 μm (Figure 2). The well-defined morphology suggests suitability for skin penetration and effective transdermal administration.

### 3.4 Pharmacokinetic profile of miRNA55 via different administration routes

**Figure 3:**
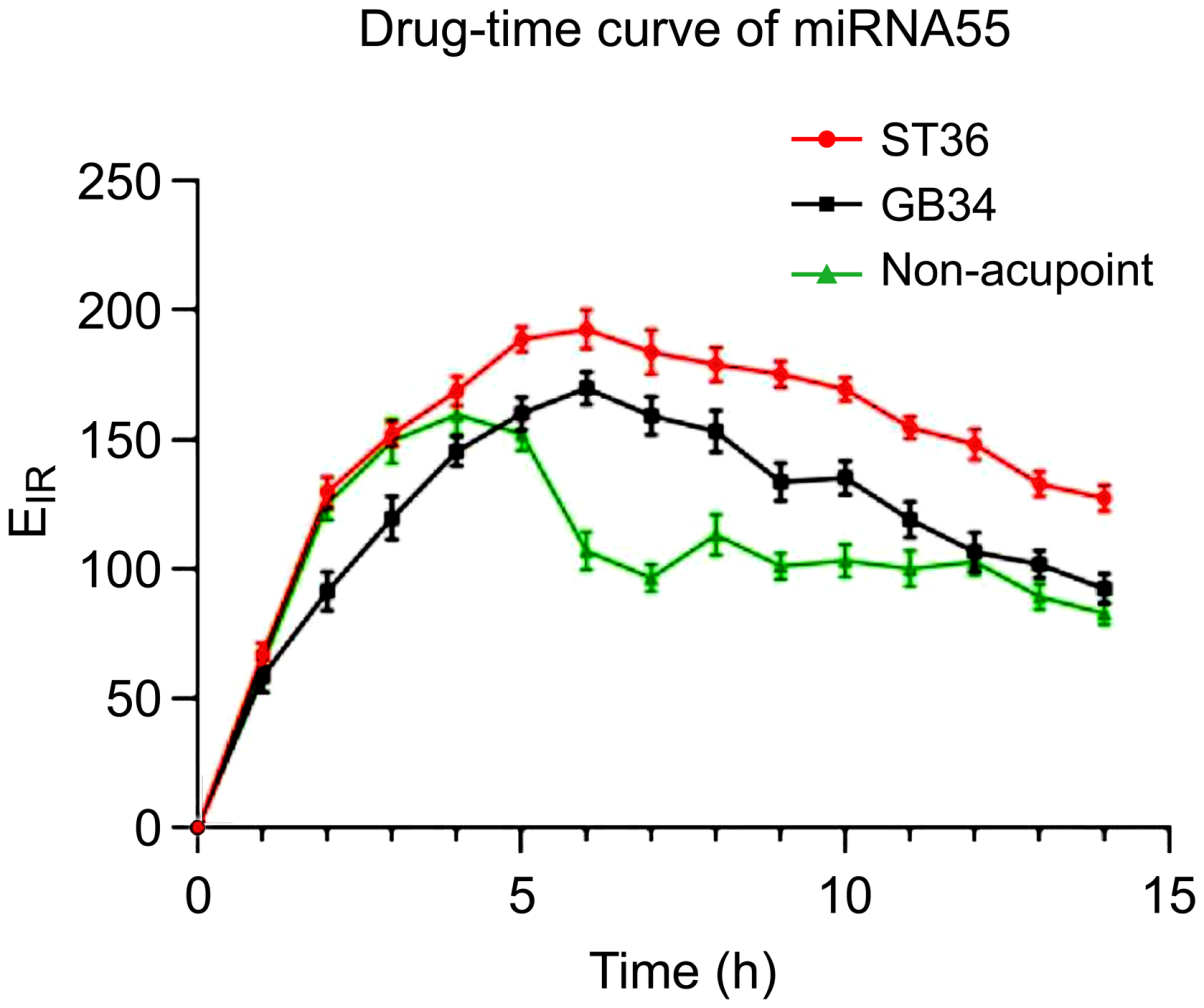
Joint cavity effusion time curves of miRNA55 under different transdermal administration routes.

The pharmacokinetic profiles of miRNA55 following transdermal administration via MNs at different anatomical sites are shown in Figure 3. Across all groups, miRNA55 exhibited measurable systemic absorption, but distinct differences were observed in the absorption rates and extent of drug exposure depending on the administration site. Among the groups, the non-acupoint administration resulted in rapid absorption, reaching peak concentration earlier than the other groups. However, this was followed by a more rapid decline, indicating unsatisfactory drug retention. In contrast, miRNA55 administration at classical acupoint (ST36) led to higher overall drug exposure and more sustained plasma concentration. The non-classical acupoint (GB34) group exhibited intermediate absorption and elimination characteristics. After reaching the peak value, the drug retention ability of the two acupoint groups was stronger than that of the non-acupoint group, which may be due to the storage effect of the same acupoint. The elimination rates of classical and non-classical points were similar; however, the blood drug concentration at classical points was still higher than that at non-classical points, which is in accordance with the theory of traditional Chinese acupuncture points. The absorption rate constant (k01) were the highest in the classical and non-acupoint groups, while elimination rates were comparable across all groups. The pharmacokinetic parameters (Table 2) supported these observations, and the area under the concentration-time curve (AUC) was the highest in the classical acupoint group, followed by the non-classical acupoint group. The non-acupoint administration group had the smallest AUC, indicating that the classical acupoint group had the best degree of drug absorption. Based on the above analysis, the same drug component (miRNA55) showed different pharmacokinetic behaviors at different drug administration sites (classical acupoints, non-classical acupoints, non-acupoints). Therefore, different acupoints had different absorption degrees for the same drug.

**Table 2:** Pharmacokinetic parameters of miRNA55 (*n* = 6).

Reference data	ST36	GB34	Non-point
AUC_0−_t/h·ng·mL ^−1^	3371.24 ± 1125.21	2846.44 ± 1021.25	2492.79 ± 1158.23
K_01__HL/h	2.85 ± 0.86	4.12 ± 0.82	1.19 ± 0.23
K_10__HL/h	6.83 ± 1.21	4.17 ± 0.95	11.44 ± 2.11
K_01_/h^−1^	0.24 ± 0.04	0.16 ± 0.02	0.68 ± 0.09
K_10_/h^−1^	0.12 ± 0.01	0.17 ± 0.01	0.06 ± 0.01
T_max_/h	6.16 ± 0.91	5.94 ± 0.83	4.33 ± 0.31
C_max_/ng·mL^−1^	192.64 ± 59.21	169.85 ± 43.22	159.63 ± 34.52

Model fitting analysis using WinNonlin 8.1 software indicated that a one-compartment model best described the pharmacokinetics of miRNA55 for all groups, as noticed by lower values of the Akaike information criterion (AIC) and Schwartz Bayes information criterion (SBC) (Table 3).

**Table 3:** Atrioventricular model fitting of miRNA55 microneedle transdermal drug delivery (*n* = 6).

Index	One-compartment model	Two-compartment model
Atrioventricular model of miRNA55 administered through ST36 AIC	80.63	84.64
Atrioventricular model of miRNA55 administered through ST36 SBC	82.55	87.83
Atrioventricular model of miRNA55 administered through GB34 AIC	88.71	92.77
Atrioventricular model of miRNA55 administered through GB34 SBC	90.62	95.97
Atrioventricular model fitting of miRNA55 through non-acupoint AIC	84.51	88.49
Atrioventricular model fitting of miRNA55 through non-acupoint SBC	86.41	91.68

### 3.5 Pharmacodynamic results

**Figure 4:**
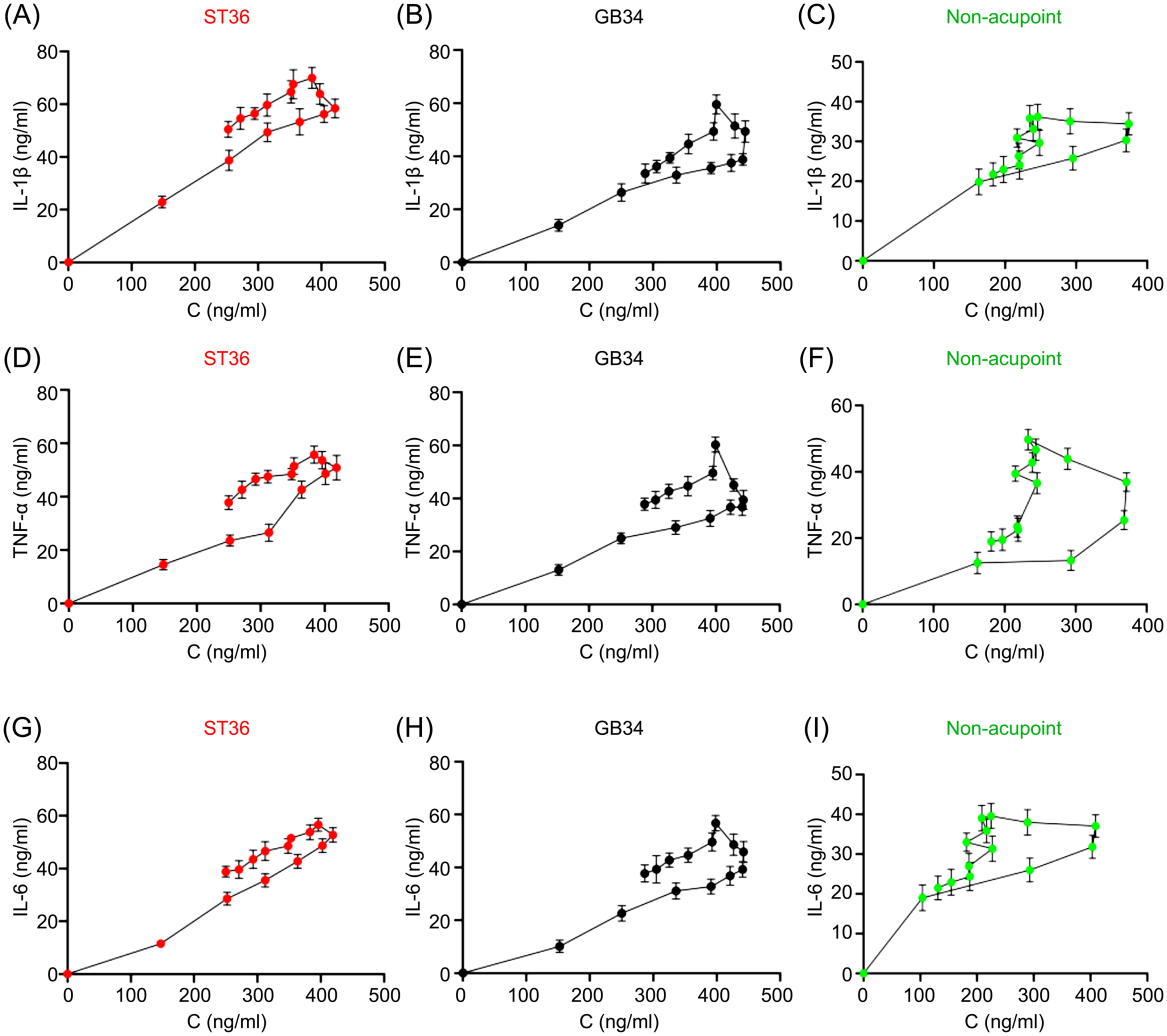
IL-1β, TNF-*α*, and IL-6 concentrations in Joint cavity effusion following administration via ST36, GB34, and non-acupoint routes.

The pharmacodynamic effects of miRNA55 were evaluated by analyzing the levels of pro-inflammatory cytokines (IL-1β, TNF-*α*, and IL-6) in the articular cavity over a 14-h period post-administration. As shown in Figure 4, the E-C (effect-concentration) relationship showed a counterclockwise hysteresis loop, indicating no direct correlation between drug inhibition and drug concentration within the 14-h timeframe. Following administration, both drug concentration and inhibitory effect initially increased and then decreased, though not simultaneously. The inhibitory effect of the drug slightly lagged behind peak drug concentration, demonstrating a delayed pharmacodynamic response, i.e., the inhibitory effect continued to rise even as drug concentration began to decrease.

### 3.6 PK/PD model fitting

PK/PD modeling was performed to quantify the relationship between miRNA55 concentrations and cytokine inhibition. The fitting parameters for miRNA55 are shown in Table 4. As shown in Table 4, among the drug administration sites, the largest Emax was noticed in the classical (ST36) acupoint administration group, while the smallest Emax was noticed in the non-acupoint administration group. This observation indicated that the acupoint group had a higher degree of drug absorption than the non-acupoint group, which is in line with the traditional Chinese acupuncture point theory. This observation also indicated that the inhibition of inflammatory factors (IL-1β, TNF-*α*, and IL-6) was enhanced. The EC_50_ was also the highest in classical acupoint group, demonstrating the advantages of the typical acupoint ST36 group. The rate constant Ke0 (for IL-1β group and TNF-*α*), representing the equilibrium between the effect compartment and the target tissue, was the lowest in in the classical acupoint group, suggesting a slower but more stable onset of action. The non-acupoint group showed the highest Ke0 for IL-6, indicating that the absorption of drugs in the non-acupoint group was slower. Typical acupoints have been proven to be the best transdermal administration site. A fitting analysis of each parameter was performed, and the fitting pharmacodynamic equations for different administration routes were obtained as $E=\frac{E_{\max}\times C_{e}}{{\mathrm{EC}}_{e50}+C_{e}}$ . The fitted PK/PD equations for IL-1β as the pharmacodynamic marker for the classical acupoint ST36 group, non-classical acupoint GB34 group, and non-acupoint group were $E=\frac{81.09C_{e}}{169.56+C_{e}}$ , $E=\frac{76.80C_{e}}{156.61C_{e}}$ , and $E=\frac{55.92C_{e}}{119.88+C_{e}}$ , respectively. For TNF-α, the fitted PK/PD equations for classical acupoint ST36, atypical acupoint GB34, and non-acupoint groups were $E=\frac{70.35C_{e}}{136.15+C_{e}}$ , $E=\frac{62.11C_{e}}{122.15+C_{e}}$ , $E=\frac{43.52C_{e}}{102.15+C_{e}}$ , respectively. The fitted PK/PD equations for IL-6 as the pharmacodynamic marker for ST36, GB34, and non-acupoint groups were $E=\frac{69.93\times C_{e}}{167.88+C_{e}}$ , $E=\frac{76.44\times C_{e}}{153.58+C_{e}}$ , and $E=\frac{49.20C_{e}}{108.52+C_{e}}$ , respectively. These results collectively support the superior pharmacodynamic performance of ST36 (classical acupoint) as a transdermal delivery site for miRNA55, consistent with TCM principles.

**Table 4:** Fitting parameters of miRNA55 with diameters of different administration routes (*n* = 6).

Reference data	Route of medication	*E*_max_/%	EC_e50_/ng·mL^−1^	*k_eo_*
IL-1β	ST36	81.09	169.56	0.49
	GB34	76.80	156.61	0.57
	Non-point	55.92	119.88	0.54
TNF-α	ST36	70.35	136.15	0.49
	GB34	62.11	122.15	0.59
	Non-point	43.52	102.15	0.54
IL-6	ST36	69.93	167.88	0.55
	GB34	76.44	153.58	0.59
	Non-point	49.20	108.52	0.66

### 3.7 Histopathological changes in synovial tissue inflammation

**Figure 5:**
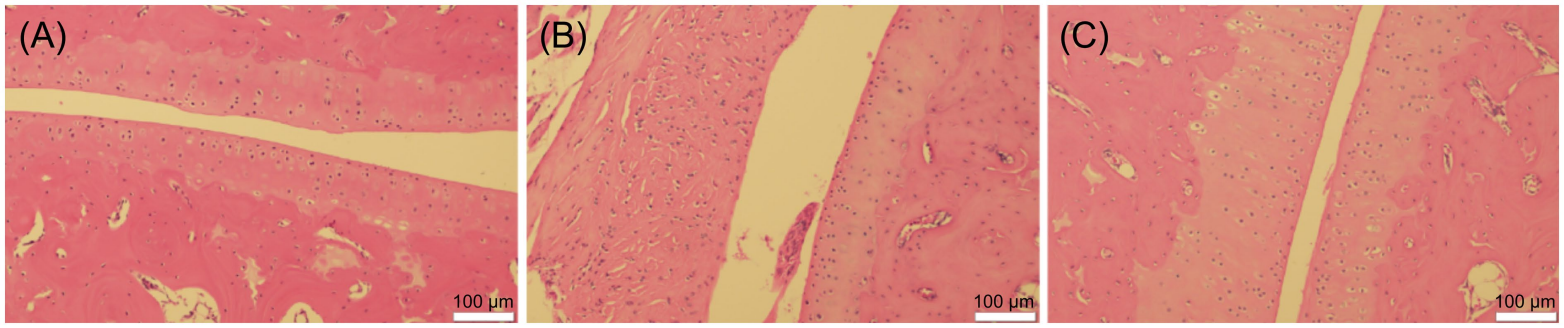
Histopathological examination of synovial membrane from rabbit ankle joint (H&E staining, ×100 magnification).

Histological examination of the ankle joint synovium revealed distinct differences among the treatment groups (Figure 5). In the ST36 group (classical acupoint), synovial tissue exhibited only occasional hyperplasia and minimal lymphocyte infiltration, indicating limited inflammatory response. The GB34 group (non-classical acupoint) showed moderate connective tissue and synovial membrane hyperplasia, and a similar degree of lymphocytic infiltration compared to that of the ST36 group. However, the non-acupoint group displayed significant pathological changes, including extensive connective tissue hyperplasia and a higher degree of lymphocyte infiltration, suggesting a higher degree of inflammation. These observations support the anti-inflammatory superiority of classical acupoint administration via miRNA55-HA MNs.

### 3.8 Expression of inflammatory marker proteins

**Figure 6:**
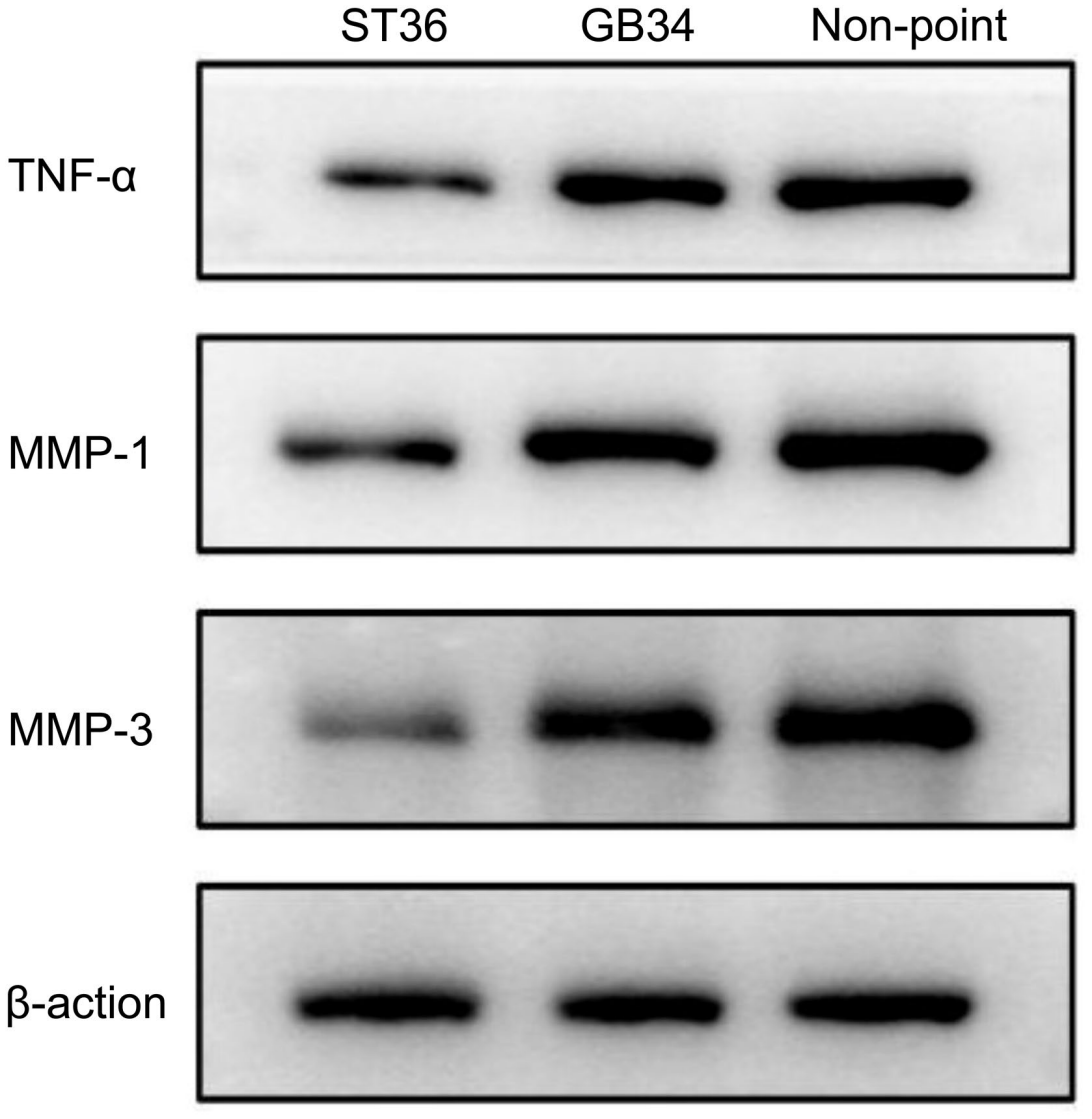
The effect of miRNA55-CLs-HA microinjections on protein expression in rabbit synovial tissue.

**Figure 7:**
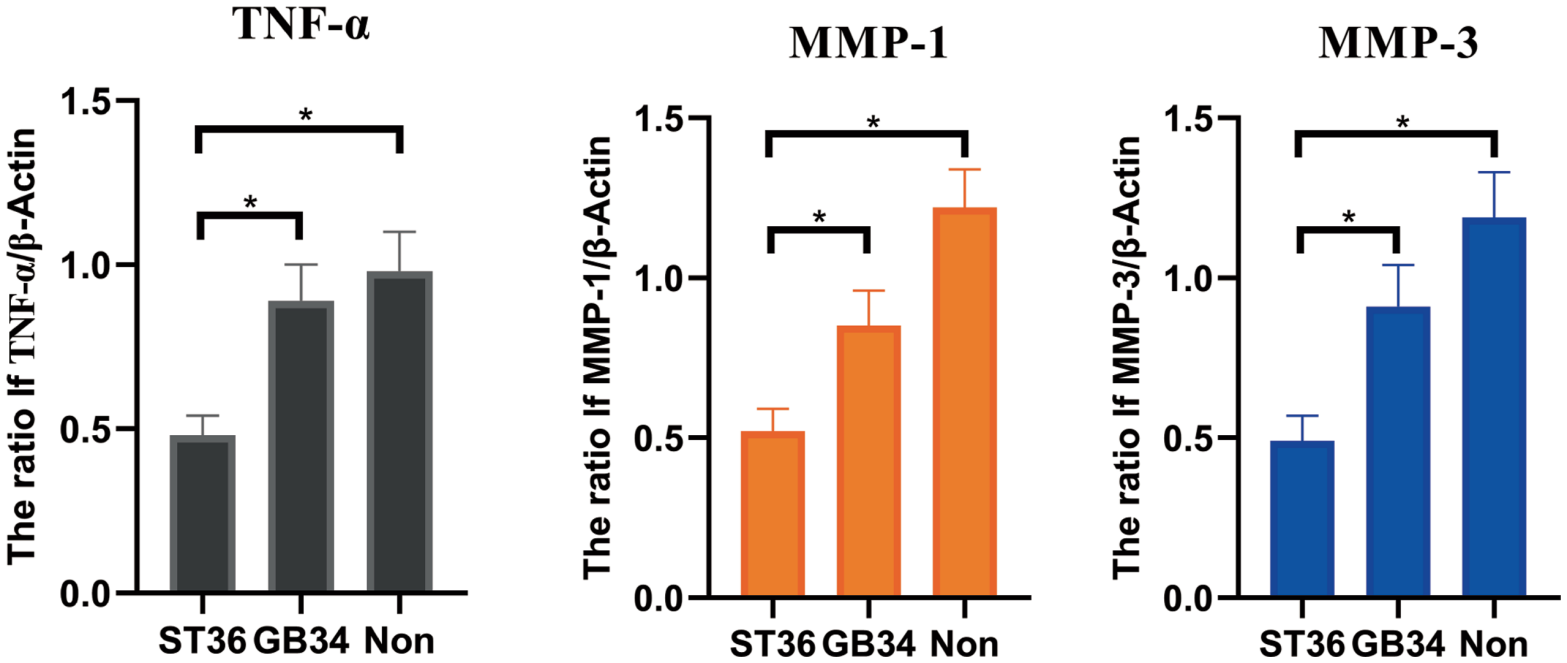
Detection of the expression of related proteins in rabbit synovial tissue analyzed by western blotting.

To further investigate the anti-inflammatory effects of miRNA55-HA MNs, protein expression of key inflammatory markers in synovial tissue was assessed by WB (Figure 6). Administration via ST36 resulted in a significant downregulation of TNF-*α*, MMP-1, and MMP-3 protein levels, in comparison to those in both the GB34 and non-acupoint groups. The non-acupoint groups showed the highest expression levels of these inflammatory markers, consistent with the histopathological findings and pharmacodynamic data. Figure 7 presents the grayscale analysis of western blotting (WB) results for the three drug administration routes, further illustrating the advantages of acupoint drug delivery and confirming the therapeutic effects of miRNA55. These results confirm that miRNA55 delivered via classical acupoint MNs not only improves drug absorption and retention but also exerts a strong inhibitory effect on inflammation-related protein expression in synovial tissue.

## 4 Discussion

RA is a chronic autoimmune disease characterized by persistent joint inflammation. It is driven by genetic and environmental factors that disrupt immune tolerance (20). In this study, we explored the use of a novel miRNA55-loaded liposomal HA-MNs system (miRNA55-CLs-HA-MNs) for transdermal drug delivery targeting RA-related inflammation. HA-based MNs offer several advantages, including enhanced skin permeability, biodegradability, and the ability to facilitate sustained drug release. Our *in vitro* results confirmed a biphasic release pattern for miRNA55, initial rapid release within the first 8 h, followed by a sustained phase. This biphasic release highlights the potential of HA as a matrix for controlled delivery. The high encapsulation efficiency (>91%) further supports the stability and efficiency of the formulation. Over a 90-day period, hyaluronic acid microneedles (HA MNs) of two sizes (5 × 5 and 10 × 10 arrays) were applied to the dorsal skin of female and male mice, respectively. The general condition, serum biochemistry, and complete blood cell count of the mice were observed and recorded. The results demonstrated that HA MNs exerted no obvious toxic effects in mice. Subsequently, the main organs of the mice were dissected and examined. No obvious morphological abnormalities were observed between the control group and the MN-treated group after 90 days of HA MN application (21).

MiRNAs have emerged as promising biomarkers and therapeutic agents in RA and act as regulators of immune balance and inflammation (22). PK and PD analyses can provide crucial insights into drug absorption and efficiency between drugs and the body. The correct application of the PK/PD model can reduce the cost of the experiment and reduce the probability of experimental failure. Thus, PK/PD models are of great significance for the evaluation of drug effects and optimization of drug regimens, providing a basis for clinical drug use, exploring the mechanism of drug action, and providing a basis for drug research (23). PK and PD analysis revealed significant differences in drug absorption and retention depending on the administration site. Specifically, drug delivery at classical acupoint ST36 showed the highest absorption (AUC), slower elimination, and prolonged retention compared with non-acupoint and non-classical acupoint sites. These findings are consistent with the theory of acupoint “drug reservoir effects,” which states that specific meridian points enhance drug bioavailability and therapeutic duration. The one-compartment model provided a better fit for miRNA55 pharmacokinetics than the two-compartment model, suggesting a uniform distribution phase following transdermal absorption. PD results were consistent with PK results. For example, the anti-inflammatory effect, as measured by IL-1β, TNF-*α*, and IL-6 inhibition in synovial fluid, was more pronounced in the ST36 group than in the GB34 and non-acupoint groups. Importantly, the Emax values for IL inhibition were the highest in the ST36 group, which supports the superiority of classical acupoint delivery for enhancing therapeutic efficacy. The observed counterclockwise hysteresis in the E–C curves suggests a delayed pharmacodynamic response, likely due to the time required for miRNA-mediated gene modulation following absorption.

Histopathological evaluation using H&E staining of synovial tissue provided further supporting evidence. For example, ST36-treated animals exhibited minimal lymphocyte infiltration and synovial hyperplasia, whereas the non-acupoint group showed significant connective tissue proliferation and inflammatory cell infiltration. WB analysis supported these morphological findings, with lower expression of inflammatory markers in the ST36 group. Our findings align with previous studies supporting the therapeutic role of acupuncture points, particularly ST36 and GB34, in treating RA. For example, Chuanyu et al. found that moxibustion ST36 could reduce MMP-9 levels in mice with RA (24), while Xiaolan et al. reported similar MMP-9 reductions following ST36 treatment. Electroacupuncture at ST36 has been shown to regulate neurotransmitters, mediate T cell apoptosis, regulate Th1/Th2 cytokines, and affect RA-related signaling pathways (25). Xian et al. demonstrated that injecting bee venom at ST36 significantly reduced the expression of IL-1β in rats (26). Ruan et al. found that embedding ST36 improved RA symptoms in patients (27), and Guangyao et al. reported its significant analgesic and anti-inflammatory effects (28). Yang et al. further revealed that acupuncture at ST36 promotes immune balance by regulating the activation of macrophages (to promote M1/M2 balance), mast cell degranulation (to initiate body self-healing), and T lymphocyte subsets and restores self-stability by apoptosis (29). Xiufang et al. found that stimulating both GB34 and ST36 acupoints in RA rats reduced arthritis symptoms and swelling (30). Clinically, Quanyi et al. noticed that the GB34 point is frequently used for RA treatment (31), and Sihui et al., through data mining, found that GB34 was applied 11 times in RA therapy, where RA is regarded as “arthralgia” in TCM (32). Together, these studies support our findings on the role of ST36 and GB34 in modulating inflammation and immune function in RA. Our findings provide mechanistic insight into how miRNA55, when delivered to specific acupoints, may produce synergistic effects through both pharmacological and acupuncture-mediated pathways.

In addition to the crucial insights provided by our study, it also has a few limitations. For example, while the rabbit model offers translational relevance, human skin permeability and acupoint response may differ. Further, although miRNA55 targets are implicated in RA-related pathways, specific gene silencing or expression changes were not evaluated in this study. Future work should aim to validate downstream gene modulation and conduct comparative studies in larger animal models or clinical trials.

In conclusion, this study demonstrates the successful development of a novel transdermal delivery system using cationic liposome-encapsulated miRNA55 loaded into HA MNs, with administration via acupoints. The integration of mRNA therapeutics with TCM principles, particularly acupoint therapy, offers a synergistic strategy to enhance drug absorption and anti-inflammatory efficacy in RA. The PK/PD results confirmed that acupoint administration (at the ST36 site) significantly improved therapeutic outcomes compared to the case of non-acupoint delivery. This study not only supports the clinical potential of miRNA-based acupoint therapy in autoimmune disease but also provides a modernized framework for the revitalization and integration of TCM into contemporary pharmaceutical applications. However, this chapter does not include longitudinal arthritis severity scores, functional assessments, X-ray findings, or structural joint evaluations, which represents a certain limitation that will be addressed in our follow-up studies.

## Data Availability

The raw data supporting the conclusions of this article will be made available by the authors, without undue reservation.

## Glossary

AIC: Akaike Information Criterion
API: Active Pharmaceutical Ingredient
AST: Aspartate Aminotransferase
BIC: Bayesian Information Criterion
CMA: Central Microdialysis Association
DEPC: Diethyl Pyrocarbonate
DOTAP: 1,2-dioleoyl-3-trimethylammonium-propane
ELISA: Enzyme-Linked Immunosorbent Assay
FLS: Fibroblast-Like Synoviocytes
FEPTubing: Fluorinated Ethylene Propylene Tubing
GB34: Yanglingquan 34 Acupoint
HA: Hyaluronic Acid
H&E: Hematoxylin and Eosin
IL-1β: Interleukin-1 beta
IL-6: Interleukin-6
IR: Inhibition Rate
miRNA: MicroRNA
miRNA55: Specific microRNA used in the study
MMP-9: Matrix Metallopeptidase 9
NF-κB: Nuclear Factor Kappa-light-chain-enhancer of Activated B cells
NLRP3: NACHT, LRR, and PYD Domains-Containing Protein 3
OD: Optical Density
PD: Pharmacodynamics
PK: Pharmacokinetics
RA: Rheumatoid Arthritis
RNA: Ribonucleic Acid
SBC: Schwarz Bayesian Criterion
ST36: Zusanli 36 Acupoint
TNF-*α*: Tumor Necrosis Factor Alpha
